# Digital transformation and the Immunization agenda 2030

**DOI:** 10.2471/BLT.25.293629

**Published:** 2025-10-23

**Authors:** Mickey Chopra, Christopher Wolff, Zulfiqar A Bhutta, Francesco Checchi, Mira Johri, Thabani Maphosa, Barni Nor, Katherine L O'Brien, Ephrem Tekle Lemango

**Affiliations:** a Health, Nutrition and Population, World Bank, 1818 H Street NW, Washington, DC, 20433 United States of America (USA).; b Gates Foundation, Seattle, USA.; c Centre for Global Child Health, The Hospital for Sick Children, Toronto, Canada.; d London School of Hygiene & Tropical Medicine, London, England.; e École de Santé Publique, Université de Montréal, Montréal, Canada.; f Gavi, The Vaccine Alliance, Geneva, Switzerland.; g Swedish International Development Cooperation Agency, Stockholm, Sweden.; h Immunization, Vaccines and Biologicals Department, World Health Organization, Geneva, Switzerland.; i Health Section, United Nations Children’s Fund, New York, USA.

Digital transformation is the intentional, systematic implementation of integrated digital applications that change how governments plan, execute, measure and monitor programmes. This transformation can accelerate progress towards the *Immunization agenda 2030*, which aims to ensure that everyone, everywhere, at every age, fully benefits from vaccines.^1^ Here we describe how digital transformation can help achieve equitable immunization outcomes, and outline recommendations for governments and global partners to ensure that children in low- and middle-income countries benefit fairly.

Digital transformation can facilitate a child’s journey to full immunization.^2^ Personalized alerts and reminders improve parents’ knowledge and awareness, and help them plan and prepare for visits. At point of service, electronic immunization registries, electronic child health records and geospatial mapping of both services and communities help health workers understand which children are missing vaccines or timely vaccination, and where outreach is needed. Electronic logistics management information systems, digital job aids and interactive online training can improve quality of care, and digital and online tools can inform health workers about patients after care. These applications are widely used in well-resourced health systems. Encouragingly, the expansion of mobile internet, falling operational costs and offline-operated applications bring digital transformation within reach of programmes in less well-resourced countries too. 

Research suggests that organizational transformations often fail because they are not sufficiently comprehensive.^3^^,^^4^ Lessons from the digitalization of primary health care in low- and middle-income countries align with this finding.^5^ While digital solutions have been tested in countries of all income levels, many of these solutions have failed to scale and achieve their potential for improving immunization outcomes.^6^^,^^7^

The equity reference group for immunization^8^ is an action-oriented think tank working to generate new ideas and consolidate effective approaches to improve immunization equity. During its May 2024 meeting on digital transformation, the group highlighted the following points for discussion and action.

Digital public infrastructure is a foundation and catalyst for the digital transformation of primary health care. Digital identity, personal records, online government services, digital payments and data exchange systems are all building blocks for efficient, equitable and integrated services. Investments in digital public infrastructure for primary health care benefit children far beyond immunization.

Legal identity secures access to services and protection, and digital tools supporting birth registration facilitate improved immunization coverage. For example, when birth notification triggers the set-up of a personal digital immunization record, health workers know who to vaccinate before the child’s first contact with services. Birth registration also improves estimates of the number and distribution of children to vaccinate, and informs health workers about children who need other services.

A newborn whose electronic immunization record is populated with personally identifiable information benefits because health workers can retrieve their records through unique identifiers or demographic details, generate lists of unvaccinated children and remind parents to bring them for vaccination. Community health workers (CHWs) who identify children during home visits and other community activities can refer them for vaccination through an electronic immunization registry or electronic child health record. If parents do not own a phone, facility staff can send reminders to the local CHW.

Furthermore, with a national electronic immunization record, a child can be followed up anywhere within the country and referred electronically from one health facility to another. Even if a child presents for care for another purpose (such as curative care), the receiving health worker can continue vaccination confident of the doses the child has already received or missed. 

Vaccination status checks and catch-up vaccination at school start, during community outreach and during supplementary immunization activities and campaigns become logistically more feasible with electronic records. An electronic record also facilitates life-course vaccinations, which are administered later in life, because it is permanent and faster to review than facility-based and home-based records. These records can help predict the size and distribution of target populations for life-course doses and enable patient reminders regarding later doses.

Some countries require proof of vaccination for children to access daycare and education, and evidence of other vaccinations is often required for international travel. Long-term safe-keeping of home-based records is challenging, while digital records and certificates are traceable and shareable.

Under-vaccinated children can be reached when CHWs and facility-based providers providing other services collaborate and communicate around individual children in the same electronic child health records. For example, registers that record household asset data for social protection programmes enable monitoring of vaccination coverage by socioeconomic status such as household income, ethnicity and religion.

The organization of vaccination sessions is improved (leading to shorter wait times, reduced vaccine waste) when clinics can use registers to schedule children for vaccination on different days. Service provision is personalized when the same provider can weigh the child, check vaccination history, vaccinate, record the dose and counsel the caregiver in one sequence as opposed to sessions organized as production lines, forcing caregivers to stay in the clinic for entire sessions.

Many low- and middle-income countries depend on frequent supplementary immunization activities and campaigns to maintain population immunity, for example, to reduce the incidence of measles. Typically, children’s vaccination status is not checked during campaigns, a practice that wastes vaccine on already immune children and exposes them to the risk of adverse events. National electronic immunization records could transform how measles campaigns and supplementary immunization activities are conducted by enabling on-site confirmation of vaccination status. Digital records also enable low-cost and non-invasive community surveys to estimate population immunity in a geographic area or within a socioeconomic group even before deciding to conduct a supplementary immunization activity.

Malaria vaccination contributes to mortality reduction, particularly when combined with treated bed nets, indoor spraying, presumptive treatment and rapid diagnostic testing. Classification of malaria risk by residence and documentation of malaria preventive measures in children’s digital records will help CHWs ensure that children receive the full range.

Successful digital transformation depends on interoperability across tools, programmes and service providers, and the establishment of reliable, consolidated sources for basic information on beneficiaries, providers, facilities and commodities. Governments and their partners must replace the proliferation of standalone digital applications with a vision for digital transformation of public health services that enables functional integration of programmes and effective use of data for decision-making. Doing so will require political commitment, meaningful cross-functional collaboration across global health initiatives and donors, and investments in interoperability and digital public infrastructure.

Digital transformation must also include strategies to promote demand. Direct communication with parents in the form of alerts, reminders and information helps overcome the intention–action gap. Social listening, surveys and feedback on the quality of services stimulate community participation in the organization of immunization. Trusted and responsive online information sources, and active detection and response to misinformation in social media build trust and demand.

Artificial intelligence (AI) can also improve programme performance. AI is currently used sporadically and inconsistently for immunization programming, but case studies demonstrate its utility in identifying and targeting the unreached, identifying critical service bottlenecks, combating misinformation and optimizing task management through innovative applications.^9^ Additional strategic applications include analysing population-level data, predicting service needs and spread of disease, identifying barriers to immunization, and enhancing nutrition and health status assessments via mobile technology. Given the proliferation of AI, exploring how it can benefit immunization programming is timely, with a focus on improving practices and standards on data quality, privacy, security and ethics.

A global blueprint and increased investments in digital public infrastructure are critical starting points to enable governments to develop digital health roadmaps and build the foundation needed for digital transformation. The blueprint, based on the *Lusaka Declaration* principles,^10^ should be the backbone for country support and for developing a common vision for digital transformation of primary health-care programmes. The blueprint should outline technical and operational principles and key first steps, build on work already underway and recognize fast healthcare interoperability resources,^11^ as the global standard for exchange of health data. Governments and immunization partners should develop country-specific roadmaps using building blocks and interoperable capabilities that drive integration of immunization data with other primary health-care programme data. Furthermore, governments in collaboration with their partners can support digital transformation by pooling resources for digital public infrastructure, bolstering local talent and expertise, and shaping robust regional and local markets for technical services and technologies.

Digital transformation efforts must aim to reduce existing equity gaps within and between countries. Primary health-care digitalization in low- and middle-income countries currently lags behind that of high-income countries, with a few notable exceptions, resulting in a digital divide that hinders development. However, because low- and middle-income countries are less constrained by earlier investments, they have an opportunity to advance to more effective, less expensive and more user-friendly solutions and infrastructure. Moving forward, all digital solutions should be tailored to meet local needs and built on human-centred design principles to benefit all stakeholders, including health workers and beneficiaries.

In conclusion, digital transformation is a unique opportunity to address many longstanding challenges in immunization. Given stalled progress in vaccination coverage and reductions in financing, now is the time for bold, new approaches. Technological developments, connectivity and reduced costs increase the feasibility of digital transformation in low- and middle-income countries. Stakeholders have a strong common interest in digital health solutions, and should embrace digital transformation as an enabler for achieving the ambitious *Immunization agenda 2030* goals. Prioritizing investments in digital public infrastructure, cross-functional collaboration and the power of case-based data and AI analytics can protect more children from vaccine-preventable diseases and promote immunization equity. 
